# Antimicrobial resistance prediction by clinical metagenomics in pediatric severe pneumonia patients

**DOI:** 10.1186/s12941-024-00690-7

**Published:** 2024-04-15

**Authors:** Mingyu Gan, Yanyan Zhang, Gangfeng Yan, Yixue Wang, Guoping Lu, Bingbing Wu, Weiming Chen, Wenhao Zhou

**Affiliations:** 1 Center for Molecular Medicine, Children’s Hospital of Fudan University, National Children’s Medical Center, Shanghai, 201102 People’s Republic of China; 2 Department of Neonatology, Second Affiliated Hospital and Yuying Children’s Hospital of Wenzhou Medical University, Wenzhou, Zhejiang 325000 China; 3 Department of Critical Care Medicine, Children’s Hospital of Fudan University, National Children’s Medical Center, Shanghai, 201102 People’s Republic of China; 4 Guangzhou Women and Children’s Medical Center, Guangzhou Medical University, Guangzhou, 510005 China

**Keywords:** Metagenomic next-generation sequencing, Antimicrobial resistance, Pediatric, Severe pneumonia

## Abstract

**Background:**

Antimicrobial resistance (AMR) is a major threat to children’s health, particularly in respiratory infections. Accurate identification of pathogens and AMR is crucial for targeted antibiotic treatment. Metagenomic next-generation sequencing (mNGS) shows promise in directly detecting microorganisms and resistance genes in clinical samples. However, the accuracy of AMR prediction through mNGS testing needs further investigation for practical clinical decision-making.

**Methods:**

We aimed to evaluate the performance of mNGS in predicting AMR for severe pneumonia in pediatric patients. We conducted a retrospective analysis at a tertiary hospital from May 2022 to May 2023. Simultaneous mNGS and culture were performed on bronchoalveolar lavage fluid samples obtained from pediatric patients with severe pneumonia. By comparing the results of mNGS detection of microorganisms and antibiotic resistance genes with those of culture, sensitivity, specificity, positive predictive value, and negative predictive value were calculated.

**Results:**

mNGS detected bacterial in 71.7% cases (86/120), significantly higher than culture (58/120, 48.3%). Compared to culture, mNGS demonstrated a sensitivity of 96.6% and a specificity of 51.6% in detecting pathogenic microorganisms. Phenotypic susceptibility testing (PST) of 19 antibiotics revealed significant variations in antibiotics resistance rates among different bacteria. Sensitivity prediction of mNGS for carbapenem resistance was higher than penicillins and cephalosporin (67.74% vs. 28.57%, 46.15%), while specificity showed no significant difference (85.71%, 75.00%, 75.00%). mNGS also showed a high sensitivity of 94.74% in predicting carbapenem resistance in *Acinetobacter baumannii*.

**Conclusions:**

mNGS exhibits variable predictive performance among different pathogens and antibiotics, indicating its potential as a supplementary tool to conventional PST. However, mNGS currently cannot replace conventional PST.

**Supplementary Information:**

The online version contains supplementary material available at 10.1186/s12941-024-00690-7.

## Introduction

Antimicrobial resistance (AMR) poses a significant threat to the life and health of children [1]. The increasing rates of AMR associated with respiratory infections, resulting from the excessive or inappropriate use of antibiotics, have become a growing clinical concern [2]. The rational use of targeted antibiotic treatment plays a crucial role in reducing AMR and improving patient recovery rates [2]. However, this relies on the accurate identification of pathogenic microorganisms and AMR. Different methods are available for AMR detection or prediction, ranging from traditional (gold standard) culture-based techniques to PCR-based molecular detection and, more recently, sequencing-based methods [3, 4]. The gold standard phenotypic susceptibility testing (PST), which requires positive culture growth before conducting drug susceptibility testing, is typically time-consuming [5]. Sequencing-based drug susceptibility prediction has showed good performance in pathogens such as *Staphylococcus aureus*, providing a theoretical basis for utilizing sequencing to detect AMR [3, 6, 7]. However, whole-genome sequencing typically requires pure bacterial cultures, which are challenging to obtain in clinical practice.

In recent years, metagenomic next-generation sequencing (mNGS) has been developed to directly detect microorganism nucleic acids in clinical samples, possessing the ability to simultaneously detect microorganisms and resistance genes or mutations [8–10]. mNGS has been proven to exhibit much higher sensitivity than traditional culture methods for the detection of pathogenic microorganisms in bloodstream infections, central nervous system infections, respiratory tract infections, and other conditions [8, 10, 11]. Additionally, mNGS has been widely applied in clinical settings to detect pathogenic microorganisms in patients with various syndromes [12–14]. Some proof-of-concept studies have demonstrated the ability of mNGS to detect resistance genes in clinical samples [15–17]. However, there is a lack of understanding regarding the accuracy of AMR prediction through mNGS testing in a clinical setting, making it difficult to provide theoretical support for clinical antibiotic decision-making.

This study retrospectively compared the detection of resistant genes using mNGS and PST in children with severe pneumonia. The accuracy of mNGS in predicting drug resistance was evaluated.

## Materials and methods

### Patients’ enrollment and sample collection

We retrospectively enrolled pediatric patients with severe pneumonia in the Pediatric Intensive Care Unit (PICU) of Children’s Hospital of Fudan University between May 2022 and May 2023.

The inclusion criteria were as follows: (1) patients were diagnosed as severe pneumonia based on the clinical guidelines [18, 19]; (2) bronchoalveolar lavage fluid (BALF) tested for mNGS targeting DNA/RNA and (3) culture.

The exclusion criteria were as follows: (1) Nonbacterial infections; (2) age < = 28 days; (3) contraindications to fiberoptic bronchoscopy; (4) BALF without phenotypic susceptibility test; (5) BALF without mNGS drug resistance gene/mutation test.

### Culture and phenotypic susceptibility test

Culture and strain identification were performed using a VITEK2 COMPACT automated ID/AST instrument (bioMérieux, France), as per the manufacturer’s instructions. The Kirby–Bauer method was used to test drug susceptibility, following the Clinical and Laboratory Standards Institute (CLSI) guidelines [20].

### Metagenomics next generation sequencing

The mNGS method for diagnosing pneumonia was implemented following a standardized operating procedure. In brief, 1 mL of BALF sample was centrifuged at 12,000 × g for 5 min to collect the microorganism and human cells. Subsequently, 50 µL of the precipitate underwent host nucleic acid depletion using 1 U of Benzonase (Sigma) and 0.5% Tween 20 (Sigma), followed by a 5-minute incubation at 37 °C. The reaction was halted by adding 400 µL of terminal buffer. A total of 600 µL of the mixture was then transferred to new tubes containing 500 µL of ceramic beads for bead beating using a Minilys Personal TGrinder H24 Homogenizer (Tiangen, China). Next, nucleic acid was extracted from 400 µL of pretreated samples and eluted in 60 µL of elution buffer using a QIAamp UCP Pathogen Mini Kit (Qiagen, Hilden, Germany). The extracted DNA was quantified using a Qubit dsDNA HS Assay Kit (Invitrogen, USA).

For total RNA extraction, a QIAamp Viral RNA Kit (Qiagen, Hilden, Germany) was used, followed by removal of ribosomal RNA using a Ribo-Zero rRNA Removal Kit (Illumina). cDNA was synthesized using reverse transcriptase and dNTPs (Thermo Fisher Scientific, San Francisco, USA). DNA/cDNA libraries were constructed using the KAPA low throughput library construction kit (KAPA Biosystems, USA) according to the manufacturer’s instructions. A 750-ng aliquot of library from each sample was subjected to hybrid capture-based enrichment of microbial probes through one round of hybridization (SeqCap EZ Library, Roche, USA). Probes were designed using the CATCH pipeline with default parameters based on pathogen genomes and drug resistance genes listed in additional table S1.

The quality of the libraries was assessed using the Qubit dsDNA HS Assay kit (Invitrogen, USA) followed by the High Sensitivity DNA kit (Agilent) on an Agilent 2100 Bioanalyzer. Library pools were then loaded onto an Illumina Nextseq CN500 sequencer for 75 cycles of single-end sequencing, generating approximately 20 million reads for each library.

To ensure internal controls, DNA and RNA controls were added to the samples at a concentration of 10^4^ copies/mL. These controls included a DNA phage (Escherichia coli bacteriophage T1, ATCC 11,303-B1) and an RNA phage (Escherichia coli bacteriophage MS2, ATCC 15,597-B1). The concentrations of the controls were selected to yield 10 RPM (reads per million sequencing reads) or higher in clinical samples, where the host cell ranged from 10^3^ copies/mL to 10^7^ copies/mL. Negative controls consisted of Hela cells with 10^5^ cells/mL and sterile deionized water, which were processed alongside each batch using the same protocol. Sterile deionized water was also included as a non-template control during extraction alongside the specimens.

### Sequencing data analysis

Data analysis procedure was followed our previous pipeline [14]. The raw sequencing reads underwent initial steps of deduplication, trimming, and quality filtering. Trimmed reads were subsequently aligned to the human reference genome to eliminate human reads. Taxonomic classification of microorganisms was conducted on the remaining reads using Centrifuge (v1.0.3). To exclude potential contaminants, the number of reads for each microorganism was compared to the number observed in the negative control.

### Statistics

Data analysis was employed using R (4.1.0) software. Culture and phenotypic susceptibility test were served as the gold standard to evaluate the performance of mNGS pathogen detection and drug resistance prediction respectively. True positive (TP), false negative (FN), true negative (TN), false positive (FP), sensitivity, specificity, positive predictive value (PPV), negative predictive value (NPV) of mNGS were calculated.

## Results

### Patient characteristics

**Fig. 1:**
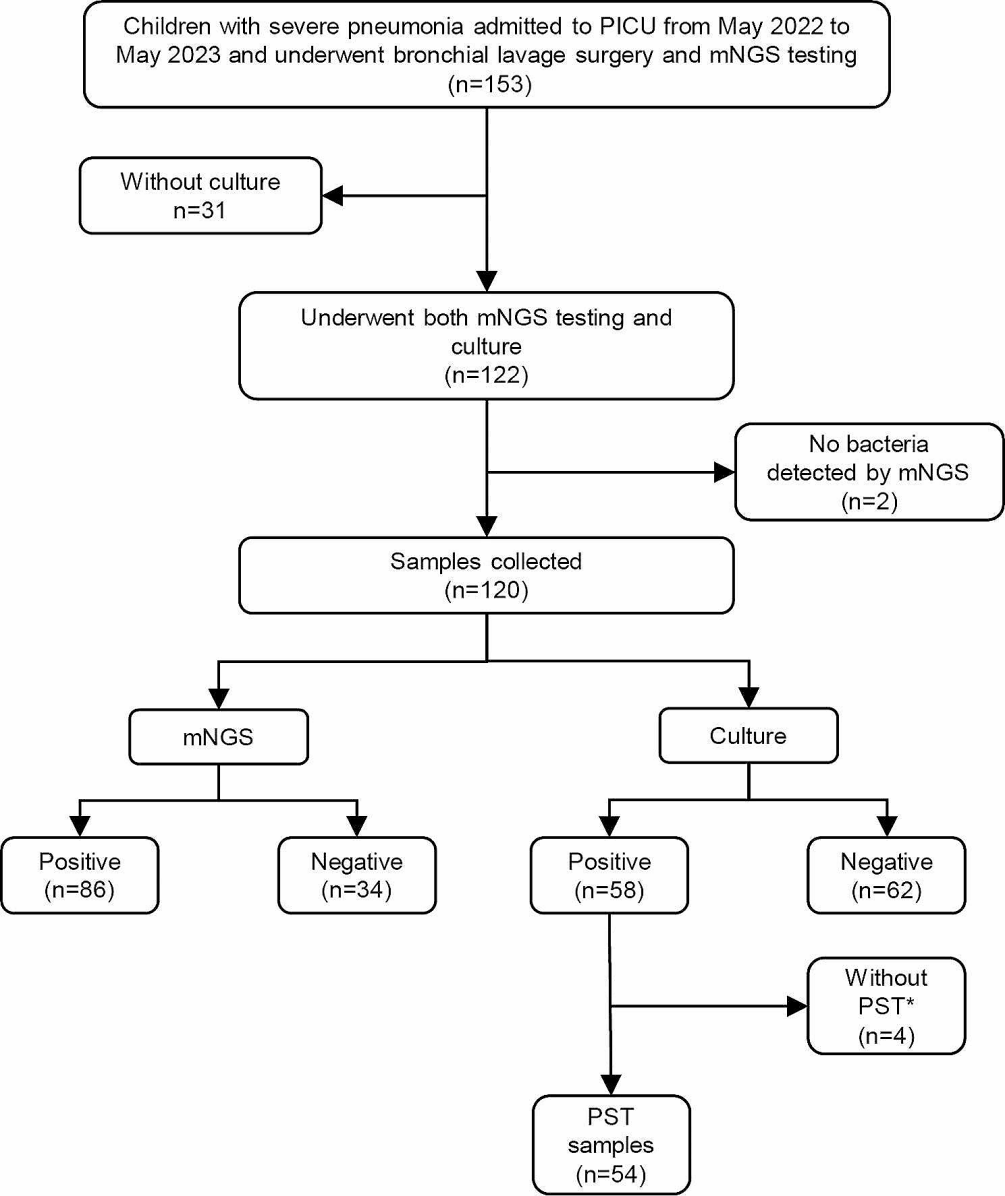
Workflow of this study

We retrospectively enrolled 120 patients who met the criteria during a one-year period (Fig. 1). Of the 120 patients, 74 (62%) patients were male. The median age was 5.5 years, half of the patients aged 6–18 years (60/120). Mechanical ventilation was administered to 97 (81%) patients. (Table 1). Most of the patients (103, 86%) have underlying diseases, such as immunodeficiency and seizures. Among the 43 immune suppressed patients, immunodeficiency (15, 13%) and hematological malignancy (10, 8.3%) were most prevalent (Table 1).

**Table 1:** Characteristics of patients

Characteristics	*N* = 120
Age	
Mean (SD)	5.99(4.41)
Median (IQR)	5.50 (2.00, 10.00)
Range	0.13, 15.00
**Sex**	
Male	74(62%)
Female	46(38%)
**Mechanical ventilation**	
Yes	97(81%)
No	23(19%)
**Immune condition**	
Immunosuppressed	43(36%)
Immunodeficiency	15(13%)
Transplant	2(1.7%)
Long-term steroids	8(6.7%)
Chemotherapy	8(6.7%)
Hematological malignancy	10(8.3%)
Nonimmunosuppressed	77(64%)
**Underlying disease**	
Yes	103(86%)
No	17(14%)
**Outcome**	
Death	10(8.3%)
Cure	110(91.7%)

### Potential pathogenic bacteria detected by culture and mNGS

**Fig. 2:**
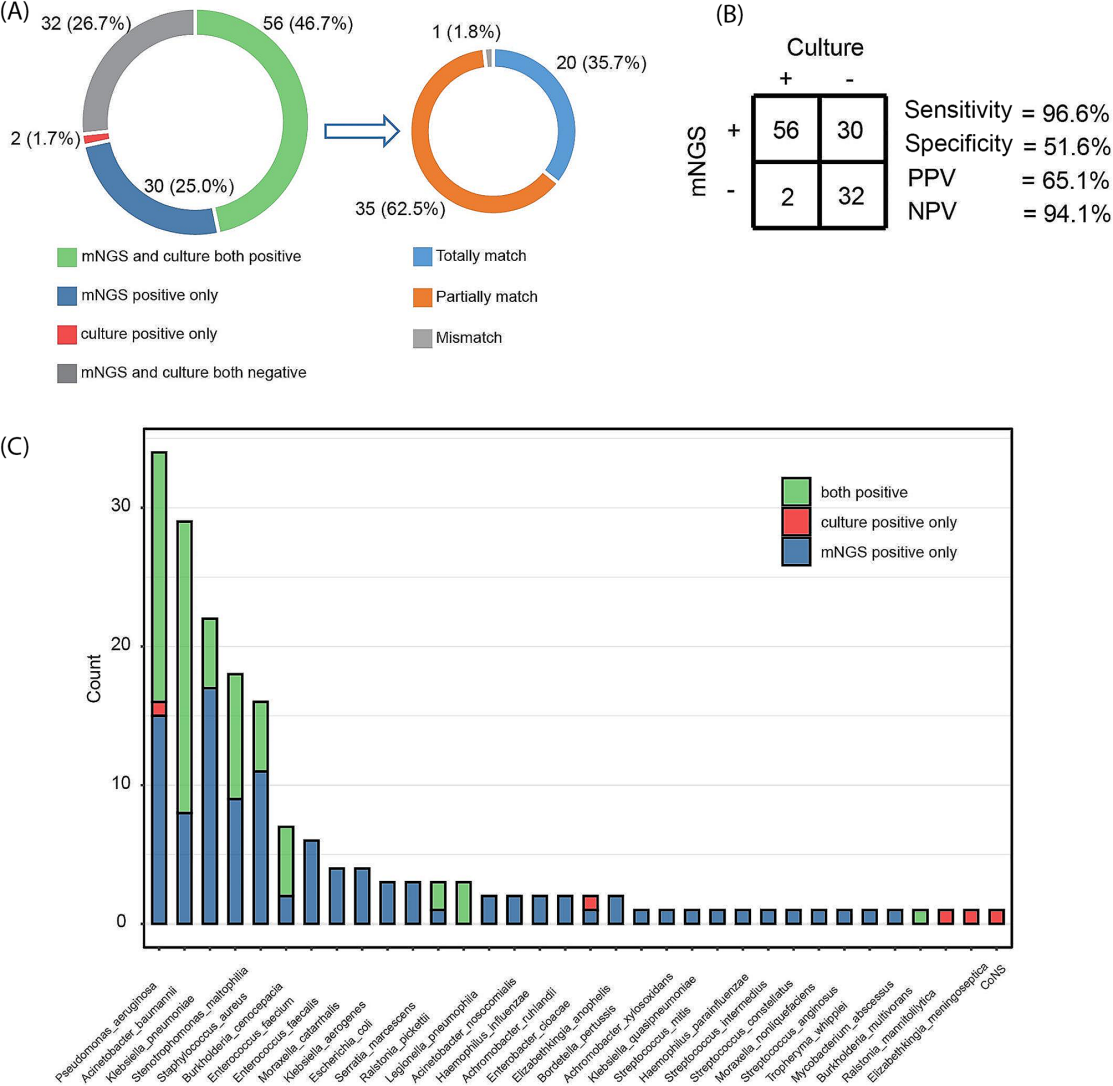
Microorganisms detected by mNGS and culture. (**A**) Accordance of detection by mNGS or culture. (**B**) The microorganism detection performance of mNGS. (**C**) Spectrum of microorganisms detected by mNGS and culture

Among the 120 BALF samples, potential pathogenic bacteria were detected positively by mNGS in 86 samples (71.7%), which was significantly higher compared to culture (48.3%, 58/120) (p-value < 0.001). Both mNGS and culture yielded positive results in 56 samples (46.7%) (Fig. 2A). Out of the 56 samples, the bacteria detected by both methods matched completely in 20 samples (36%), partially matched in 35 samples (62%), and didn’t match in only 1 sample. Bacteria were detected by mNGS alone in 30 samples (25%). Additionally, there were 2 samples in which bacteria were exclusively detected by culture.

Using culture as the standards, the sensitivity of mNGS is 96.6%, specificity is 51.6% (Fig. 2B).

In terms of bacterial types, a total of 31 bacterial species were detected by mNGS, whereas only 13 species were detected by culture. *Pseudomonas aeruginosa*, *Acinetobacter baumannii*, *Klebsiella pneumoniae*, *Stenotrophomonas maltophilia*, and *S. aureus* accounted for the majority (66.5%) of the total bacterial count. Among them, 21 species were exclusively detected by mNGS. While 3 species were detected by culture only, including *Ralstonia mannitolilytica*, *Flavobacterium meningosepticum* and *Coagulase-negative staphylococci* (Fig. 2C).

### Antibiotics resistance detected by PST and mNGS

**Fig. 3:**
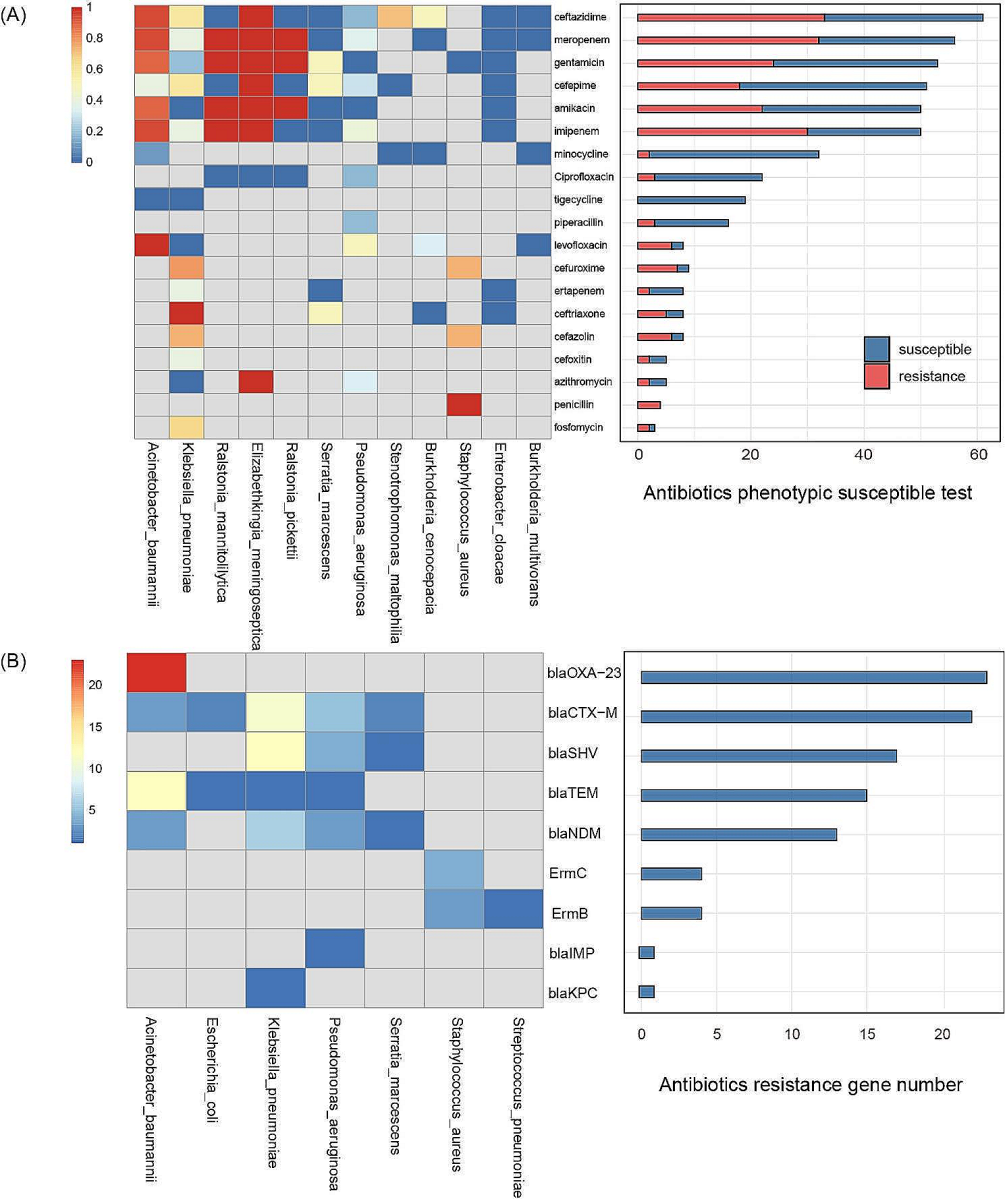
Antibiotics resistance rate and AMR genes. (**A**) left panel: heatmap of antibiotics resistance rate by PST, right panel: PST result for each antibiotic. (**B**) left panel: heatmap of AMR gene detected by mNGS, right panel: count of AMR genes detected by mNGS.

The antibiotic resistance rates of the pathogenic microorganisms are presented in Fig. 3A. Among the antibiotics with a sample count above 50 in the PST, ceftazidime, meropenem, gentamicin, cefepime, amikacin, and imipenem had resistance rates of 54.1%, 57.1%, 45.3%, 35.3%, 44%, and 60%, respectively. There were significant differences in the antibiotic resistance profiles among the top three detected pathogens. *A. baumannii* exhibited almost complete resistance to carbapenems (resistance rate of 95%), aminoglycosides (resistance rate of 89%), and third-generation cephalosporins (resistance rate of 95%). In contrast, *P. aeruginosa* showed lower resistance rates to these three classes of antibiotics, with rates of 38.2%, 0, and 17.6%, respectively. *K. pneumoniae* also demonstrated significant variation in antibiotic resistance rates, with an average resistance rate of 10% to aminoglycosides and 80% to third-generation cephalosporins. The antibiotic resistance profiles exhibited significant clustering. For example, resistance to carbapenems was mainly concentrated in *A. baumannii*, while resistance to aminoglycosides was mainly concentrated in *A. baumannii* and *P. aeruginosa*. Tetracyclines showed high sensitivity against *A. baumannii*, *K. pneumoniae*, *Burkholderia cepacia*, and *S. maltophilia*.

A total of 9 drug resistance gene were detected by mNGS (Fig. 3B). The most frequently detected gene was *blaOXA-23* gene, which is associated with carbapenem resistance of *A. baumannii* (23/100). This was followed by *blaCTX-M, blaSHV*, and *blaTEM* genes related to penicillin and cephalosporin resistance among Enterobacteriaceae and non-enterobacterial species, like *A. baumannii*, *P. aeruginosa*. The *blaNDM* gene associated with a broad spectrum of antibiotic resistance (including imipenem, meropenem, ertapenem, gentamicin, amikacin, tobramycin, and ciprofloxacin) was detected in 4 species [21]. Genes like *ermB* and *ermC*, linked to macrolide and lincosamide antibiotic resistance in *S. aureus* and *Streptococcus pneumoniae*, were detected at lower frequencies. *blaKPC* and *blaIMP* which were associated with carbapenem resistance were only detected in *K. pneumoniae* and *P. aeruginosa*, respectively.

### Performance of mNGS in antibiotics resistance prediction

Using PST as the gold standard, we assessed the performance of mNGS in predicting antibiotic resistance. We specifically evaluated its predictive ability for three classes of antibiotics: carbapenems, penicillins, and cephalosporins (Table 2). The sensitivity for predicting carbapenem resistance was higher compared to the other two categories (67.74% vs. 28.57%, 46.15%), while there was no significant difference in specificity (85.71%, 75.00%, 75.00%). Furthermore, the accuracy of carbapenem resistance prediction was also higher compared to the other two categories (75.00% vs. 57.89%, 57.14%). When examining specific pathogenic microorganisms, we first calculated the genome coverage and average depth of *A. baumannii* and *P. aeruginosa*. The genome coverage of *P. aeruginosa* was determined to be 40.38%, with an average depth of 33.65 (Table S2). For *A. baumannii*, the average genome coverage was 41.25%, with an average depth of 49.63 (Table S2). Then, we found that mNGS achieved a sensitivity of 94.74% and an accuracy of 90.00% in predicting carbapenem resistance in *A. baumannii*. However, mNGS displayed poor predictive performance for carbapenem resistance in *P. aeruginosa*, with a sensitivity of 12.5% (1/8) and a specificity of 88.9% (8/9).

**Table 2:** The performance of mNGS in the prediction of carbapenems, penicillins and cephalosporins resistance

Antibiotic type	TN	TP	FN	FP	Sensitivity (CI)	Specificity	PPV	NPV	Accuracy
Carbapenems	18	21	10	3	67.74% (48.54-82.68%)	85.71% (62.64-96.24%)	87.50% (66.54-96.71%)	64.29% (44.11-80.69%)	75.00% (60.77-85.52%)
Penicillins	9	2	5	3	28.57% (5.11-69.74%)	75.00% (42.84-93.31%)	40.00% (7.26-82.96%)	64.29% (35.63-86.02%)	57.89% (33.97-78.88%)
Cephalosporins	18	18	21	6	46.15% (30.43-62.62%)	75.00% (52.95-89.40%)	75.00% (52.95-89.40%)	46.15% (30.43-62.62%)	57.14% (44.09-69.32%)

TN: True negative, TP: True positive, FN: False negative, FP: False positive

## Discussion

mNGS detection of pathogenic microorganisms exhibits rapidity and high sensitivity. If it can also predict drug resistance, it would hold great significance for timely and effective antibiotic treatment. However, there is currently limited research evaluating the performance of mNGS in AMR prediction. Serpa et al. assessed the performance of mNGS in predicting AMR among critically ill adults with lower respiratory tract infections. They observed significant variations in the performance of mNGS across different bacteria and antibiotics [22]. The sensitivity and specificity of mNGS in predicting AMR for Gram-positive bacteria were 70% and 95%, respectively, while for Gram-negative bacteria, the sensitivity and specificity were 100% and 64%, respectively. However, the pathogen spectrum in their study differed significantly from ours, with *S. aureus*, *S. pneumoniae*, and *K. pneumoniae* being the predominant pathogens. Two other studies utilized machine learning to establish predictive models for *A. baumannii* and *P. aeruginosa*, respectively, and explored the effectiveness of mNGS in predicting AMR in clinical samples [23, 24]. The mNGS achieved a 100% concordance rate in predicting imipenem resistance in 16 cases of *A. baumannii* [23]. The predictive sensitivity for imipenem and meropenem resistance in *P. aeruginosa* was 65% and 63.2%, respectively [24].

In comparison to culture, mNGS demonstrated relatively low specificity in pathogen detection. However, this does not imply that the pathogens identified solely by mNGS were false positives. It is plausible that the low sensitivity of culture contributed to this discrepancy. The pathogens identified through mNGS may provide valuable clinical insights, as we have previously demonstrated in our work [14].

We observed significant variations in the predictive performance of mNGS for different bacteria and drugs. It showed good predictive performance for carbapenems against *A. baumannii* but poor predictive performance for carbapenems and cephalosporins against *P. aeruginosa*. The resistance mechanisms of *P. aeruginosa* are complex [25]. Besides resistance genes, mutations can also contribute to its resistance. For example, mutations in the *OprD* gene can lead to carbapenem resistance in *P. aeruginosa* [26]. In our study, we did not detect resistance mutations, which could be one of the reasons for the lower accuracy of mNGS in predicting resistance in *P. aeruginosa*. Additionally, some resistant phenotypes may not be solely attributed to resistance genes or mutations but could also result from the overexpression of certain intrinsic genes (such as the efflux pump genes *MexAB-OprM*, *MexCD-OprJ*, and *MexXY-OprM*) in *P.aeruginosa* [27]. Addressing this situation, Khaledi et al. improved the accuracy of resistance prediction by employing machine learning and transcriptome sequencing to integrate resistance genes, resistance mutations, and gene expression data [28].

The accuracy of mNGS resistance prediction largely depends on the bacterial genome coverage or the amount of effective sequencing data for the bacteria. The genetic mechanisms of bacterial resistance can be classified into horizontal transfer of resistance genes and vertical inheritance associated with mutations. Prediction of resistance is determined by detecting the presence of resistance genes, which is primarily achieved through alignment-based mapping of reads or contigs to known resistance gene databases [4]. Detection of resistance mutations requires reads to be aligned to specific loci and reach a certain depth threshold for detection, which places relatively higher demands on sequencing data volume. Prediction of sensitivity (i.e., absence of resistance genes) requires complete sequencing coverage of the bacterial genome, necessitating even higher sequencing data requirements. However, there is currently no unified threshold for genome coverage or sequencing data volume that can make resistance gene detection more reliable [29].

Due to the high proportion of host nucleic acids in clinical samples, the bacterial content in the raw sequencing data of mNGS is relatively low, which can impact the detection of resistance genes/mutations by mNGS [30, 31]. In this study, a probe hybridization capture method was employed to enrich resistance genes, aiming to improve the sensitivity of detection. In addition to hybridization capture, Crispr-Cas technology have also been utilized for specific enrichment of drug resistance genes in pathogenic microorganisms [32].

Some resistance genes are inherent to specific pathogenic microorganisms, such as the mecA gene in *S. aureus*. However, many genes are located on plasmids, which can be exchanged between different species, such as the *blaCTX-M* gene in Enterobacteriaceae. In this study, to improve the accuracy of resistance gene detection, the detected resistance genes needed to be simultaneously associated with the positive pathogenic microorganisms in the sample. Long sequence reads have advantages in linking resistance genes to host microorganisms [17, 33]. Other methods, such as Hi-C ligation, can also be used to associate resistance genes on plasmids with host chromatin [34].

The technical limitations of second-generation sequencing pose challenges for rapid detection of pathogenic microorganisms and drug-resistant genes. However, nanopore sequencing overcomes these limitations by offering real-time sequencing, eliminating the need to wait for completion and enabling concurrent data analysis. This significantly shortens the time required for pathogen detection, with nanopore applications in respiratory and fluid samples achieving detection times as short as 6 h [8, 35]. The median time for detecting microorganisms after sequencing initiation is 50 min [35]. Enrichment of microbial nucleic acids can further enhance microbial detection by increasing their proportion during real-time sequencing [8, 36]. Additionally, the combination of nanopore’s adaptive sequencing and host depletion further improves the proportion of microbial reads and detection sensitivity [31, 37]. The promising clinical applications of nanopore’s real-time sequencing highlight its importance in guiding rapid and informed antibiotic use in clinical settings.

The predictive performance of mNGS for drug resistance is highly correlated with the detection of pathogenic microorganisms. In this study, critically ill children with pneumonia in the PICU were included, and a specific spectrum of pathogenic microorganisms was detected. The top ranked pathogens were *P. aeruginosa*, *A. baumannii*, and *K. pneumoniae.* Therefore, the interpretation of the study’s conclusions needs to be considered in the context of specific populations and pathogens.

Our study has several limitations that should be acknowledged. Firstly, it is a retrospective, small-scale investigation, and therefore, the conclusions drawn from this study would benefit from further confirmation through larger sample size studies. Secondly, the absence of drug-resistant mutation detection or gene expression profiling in our analysis may have restricted the accurate assessment of drug prediction for mechanisms involving these specific forms of drug resistance. Thirdly, for certain drugs, the detection panel employed in this study only included a limited number of genes associated with drug resistance. For instance, only *aac6* and *tetA* genes were included for aminoglycoside and tetracycline resistance, respectively. Consequently, the predictive performance of mNGS for these two classes of drugs was not evaluated. These limitations highlight the necessity for future research with larger sample sizes and more advanced sequencing technologies to address the challenges encountered in this study more comprehensively and rigorously.

## Conclusions

This study explored the performance of mNGS in predicting drug resistance in children with severe pneumonia. We found significant variations in the predictive performance of mNGS among different pathogens and drugs, indicating its potential as a supplementary tool to conventional PST. However, mNGS currently cannot replace conventional PST.

### Electronic supplementary material

Below is the link to the electronic supplementary material.

Supplementary Material 1

Supplementary Material 2

## Data Availability

Not applicable.

## Abbreviations

mNGS: metagenomic next-generation sequencing
AMR: antimicrobial resistance
PST: phenotypic susceptibility testing
BALF: bronchoalveolar lavage fluid
